# New Appointment

**Published:** 1853-09

**Authors:** 


*New Appointment.*—Dr. Thomas F. Rochester, of this city, has been appointed to the chair of Theory and Practice of Medicine in the Buffalo Medical College. We know the man, and can vouch for his professional ability and moral worth, by personal association with him in the toils and sacrifices of hospital practice. Dr. R. was one of our valued assistants at Bellevue, in 1848, where he distinguished himself by ardent devotion to his duties, and signal usefulness in the midst of ship fever, with the treatment of which he acquired great familiarity and success. We congratulate him on his early appreciation by so respectable a school as that of Buffalo, and the faculty upon the acquisition of an associate who is a physician, a scholar, and a gentleman. We predict for him a career of honor and reputation, whether as a teacher or practitioner, and only regret that our city, by his removal to Buffalo, will lose one of the most promising of our young practitioners, who had endeared himself here to many friends.—*New York Med. Gaz.*

